# Supplementary material for: Magnetic Levitation Patterns of Microfluidic-Generated Nanoparticle–Protein Complexes
Source: Nanomaterials (Basel). 2022 Jul 11;12(14):2376. doi: 10.3390/nano12142376 (PMC9324036; doi:10.3390/nano12142376)
Supplement: Supplementary file 1 [file nanomaterials-12-02376-s001.zip › nanomaterials-1771758-supplementary.pdf]

nanomaterials

Supplementary Materials

Magnetic Levitation Patterns of Microfluidic-Generated Nanoparticle–Protein Complexes

Luca Digiacomo [1,†], Erica Quagliarini [1,†], Benedetta Marmiroli [2], Barbara Sartori [2], Giordano Perini [3,4], Massimiliano Papi [3,4], Anna Laura Capriotti [5], Carmela Maria Montone [5], Andrea Cerrato [5], Giulio Caracciolo, [1] and Daniela Pozzi [1,*]

[1] NanoDelivery Lab, Department of Molecular Medicine, Sapienza University of Rome, Viale Regina Elena 291, 00161 Rome, Italy; luca.digiacomo@uniroma1.it (L.D.); erica.quagliarini@uniroma1.it (E.Q.); giulio.caracciolo@uniroma1.it (G.C.)
[2] Institute of Inorganic Chemistry, Graz University of Technology, Stremayrgasse 9/IV, 8010 Graz, Austria; benedetta.marmiroli@elettra.eu (B.M.); barbara.sartori@elettra.eu (B.S.)
[3] Dipartimento di Neuroscienze, Università Cattolica del Sacro Cuore, Largo Francesco Vito 1, 00168 Rome, Italy; giordano.perini@unicatt.it (G.P.); massimiliano.papi@unicatt.it (M.P.)
[4] Fondazione Policlinico Universitario A. Gemelli IRCSS, 00168 Rome, Italy
[5] Department of Chemistry, Sapienza University of Rome, P.le A. Moro 5, 00185 Rome, Italy; annalaura.capriotti@uniroma1.it (A.L.C.); carmelamaria.montone@uniroma1.it (C.M.M.); andrea.cerrato@uniroma1.it (A.C.)
* Correspondence: daniela.pozzi@uniroma1.it
† These authors contributed equally to this work.

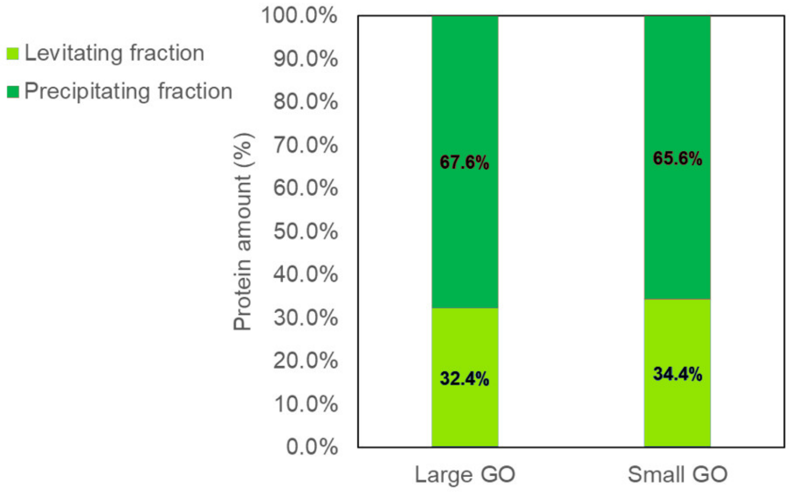

Figure S1. Large GO flakes (800 nm) and small GO flakes (300 nm) were exposed to human plasma (HP) and GO-HP complexes were injected into the MagLev device. Protein content in the levitating and precipitating MagLev fractions was quantified by the Bicinchoninic acid (BCA) assay.

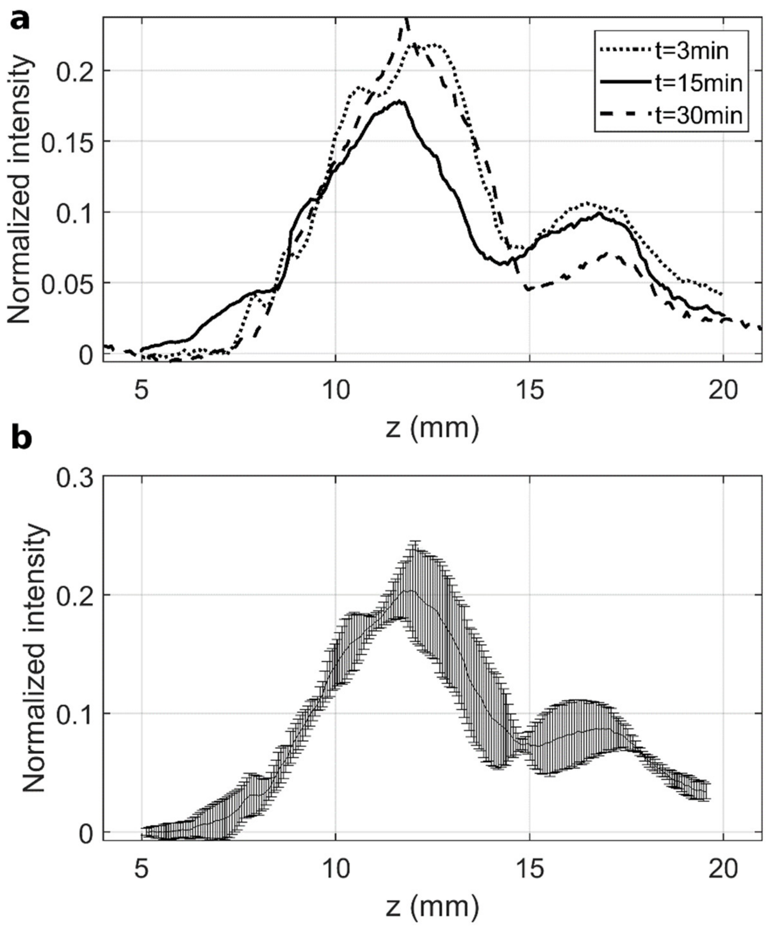

Figure S2. GO-HP samples were prepared at a 9:1 volume ratio and incubated at 26°C for 3 min, 15 min, and 30 min respectively. (a) MagLev profiles for different incubation times. (b) Average profile (solid line) and standard deviation obtained by averaging the MagLev profiles reported in panel a.

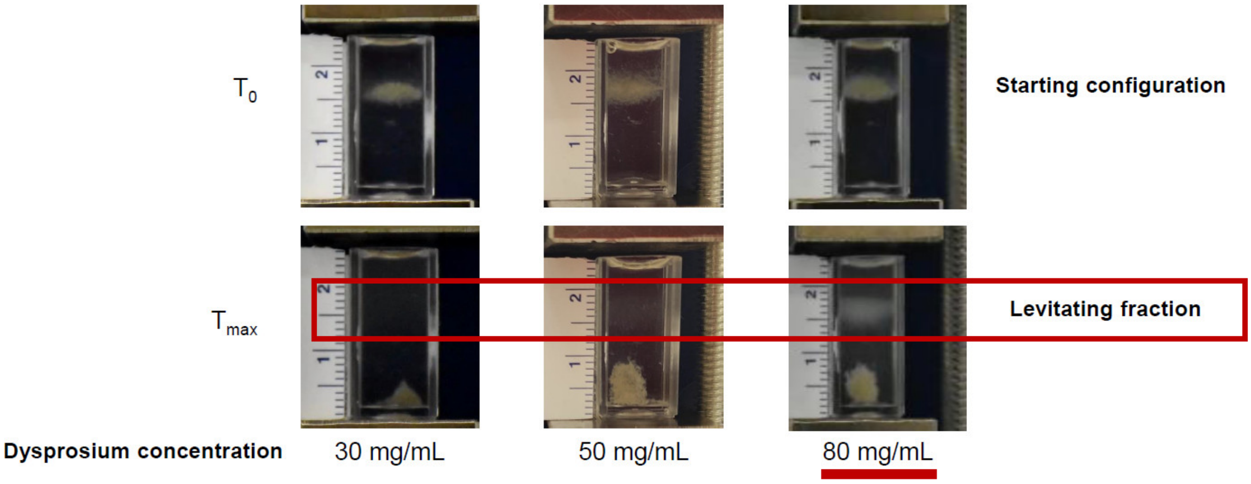

Figure S3. Starting frame and final frame of MagLev image time-series for GO-HP samples embedded in Dysprosium (III) nitrate hydrate at different concentrations. At the highest concentration, both levitating and precipitating fractions are clearly visible.

Preliminary tests of protein samples in MagLev device

The lowest protein amount returning a distinguishable MagLev readout was 2.5 µL in a total sample volume of 50 µL. However, MagLev response was not linear with sample concentration but conversely depends on manifold factors. As an instance, different macroscopic behaviors were obtained by tuning sample concentration and total sample volume (Fig. S1).

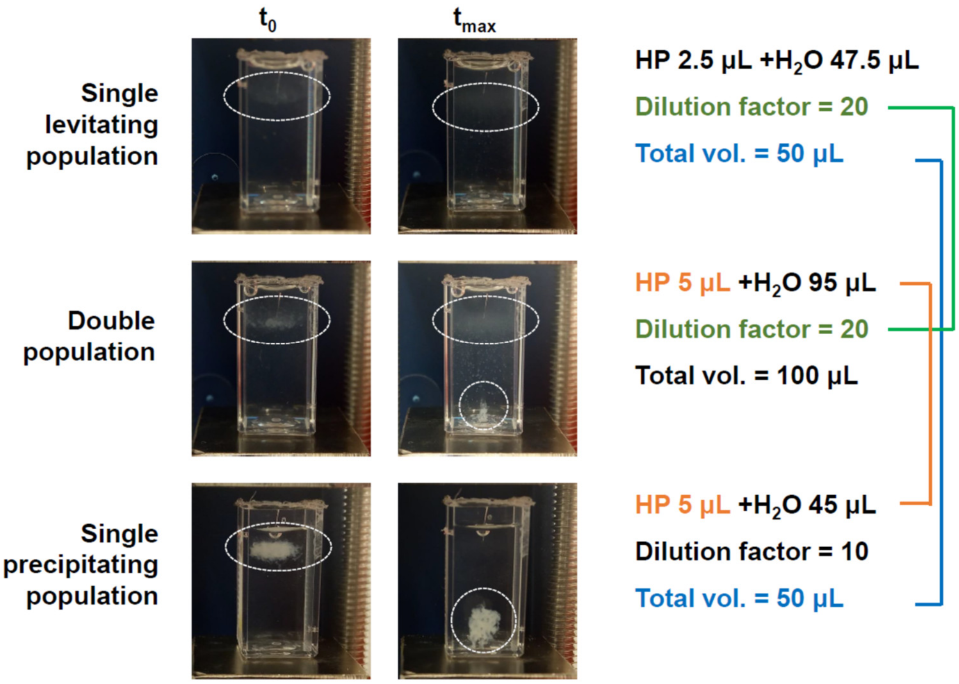

Figure S4. Representative MagLev images at t_0= 0 and t_{max}=10 min for HP samples in dysprosium (at a fixed concentration = 60 mg/mL). Different behaviors can be distinguished, *i.e.*, single levitating population, double population and single precipitating population. Total sample volume, dilution factor and absolute protein amount co-determine the resulting MagLev response.

Furthermore, to evaluate the MagLev response as a function of sample concentration, polystyrene beads were used as reference material. Beads were incubated with HP, then samples were diluted by factors 2, 3, and 5, and inserted in the MagLev device. The concentration of the paramagnetic medium and total sample volume was maintained constant. MagLev response depended on the dilution factor and exhibited different dynamics (different precipitating times), as well as single- or double- population trends.

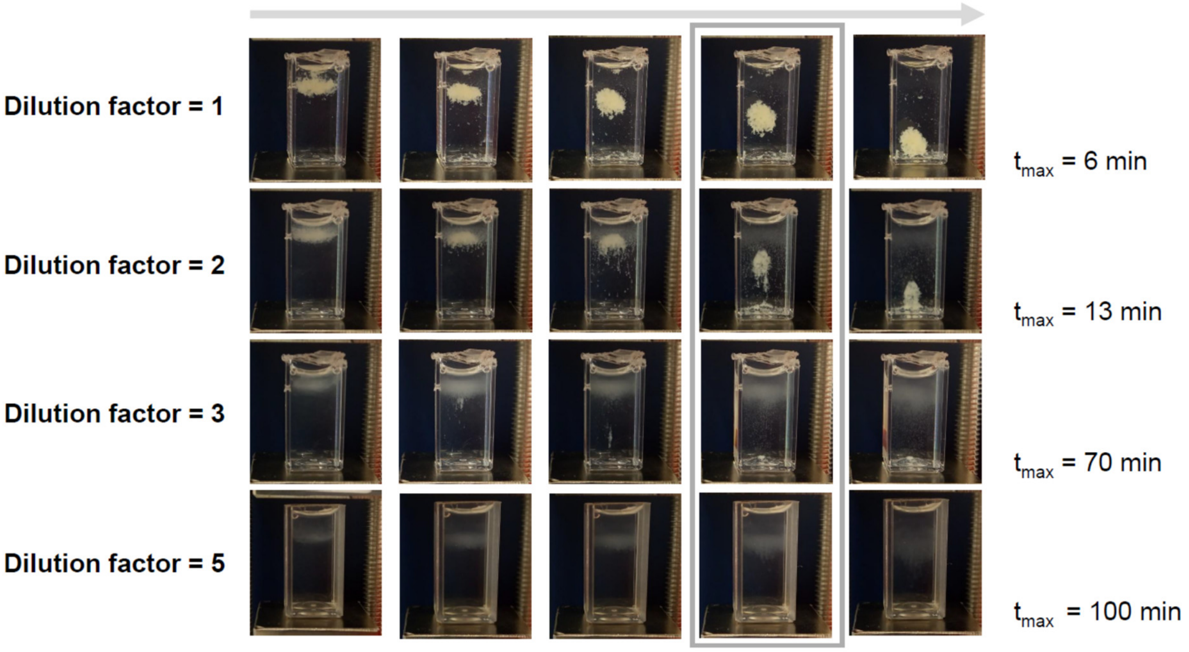

Figure S5. Representative MagLev image series for polystyrene beads-HP samples at different concentrations (all the other experimental parameters were maintained constant).

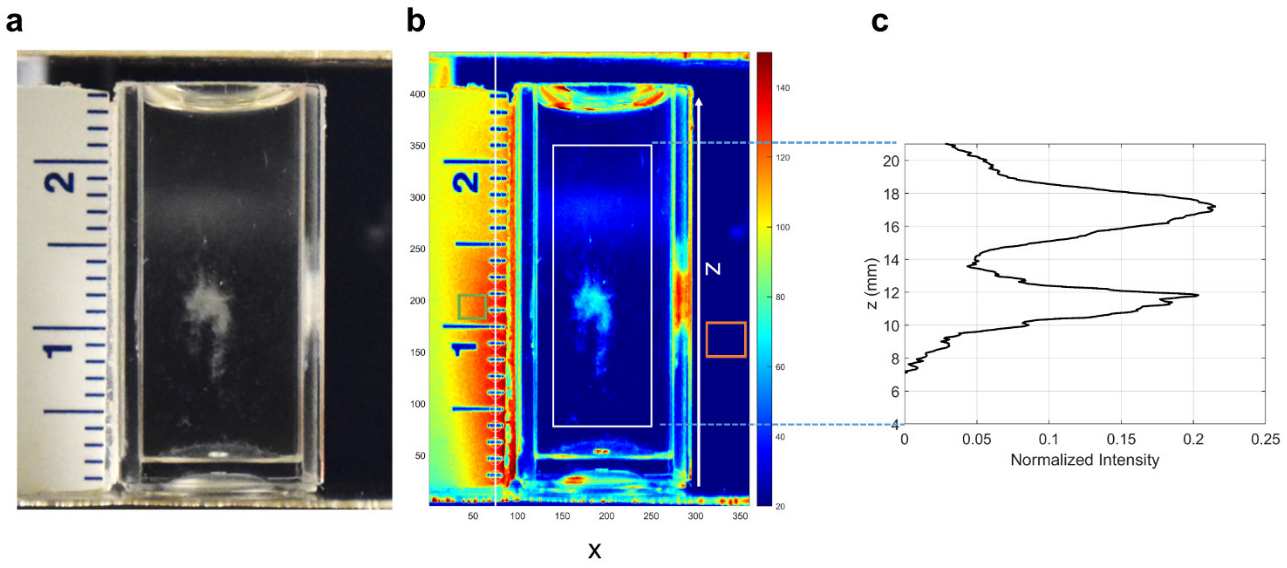

Figure S6. (a) Optical image and (b) pseudocolor image of a frame in the MagLev image time series. (c) Corresponding normalized 1-dimensional profile. Briefly, after acquiring an image time series, the intensity of each frame (optical image in Panel a and pseudocolor image in Panel b) is sampled over a region of interest (white rectangle in Panel b). Then, I(x,z) is averaged along the x-direction to obtain a 1-dimensional mean intensity profile as a function of z (Panel c). Finally, I(z) is normalized to take into account the lowest baseline background signal (average intensity over a fixed background region, *i.e.*, orange rectangle in Panel b) and the maximum intensity value of the frame (average over a fixed overexposed region, *i.e.*, the yellow rectangle in Panel c).

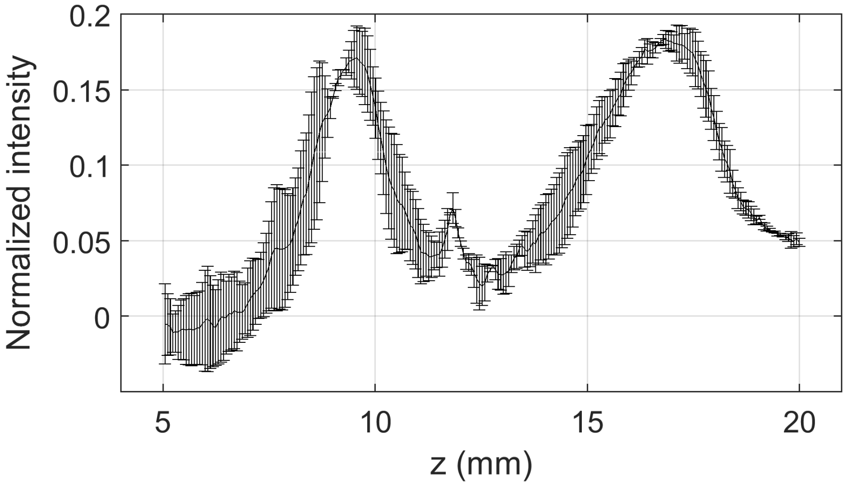

Figure S7. Representative average MagLev profile of GO-HP samples (9:1 vol/vol) incubated under static conditions. Error bars represent the standard deviation over a duplicate. The mean relative error for each of the two peaks was 16% and 9%, respectively. It was computed by averaging the relative error within a z-range spanning the full width at half-maximum region, for each peak. .

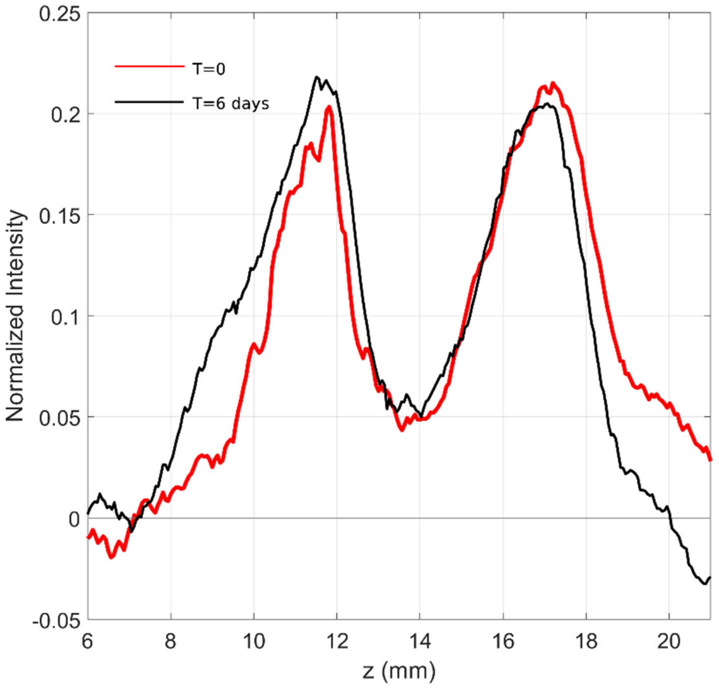

Figure S8. MagLev patterns for GO-HP samples were obtained by microfluidic incubation at a total flow rate of 3.5 µL/min and flow rate ratio of 9:1, and acquired immediately after the mixing (time=0) and 6 days later. While a slight broadening of the precipitating component was detected, these findings suggest that the mixing is completed inside the channel at the lower flow rate, or at least that it is completed in the time required to inject the volume sample into the MagLev device.

Table S1. Characterization of levitating and precipitating fractions. Protein amount in the corona formed on GO, as measured by BCA assay and total lane intensity by 1D SDS-PAGE analysis, in levitating and precipitating fractions. Protein content for the precipitating fraction was about 10 times larger than that one corresponding to the levitating counterpart.

	**Protein amount (µg)**	**Total lane intensity (a.u.)**
**Levitating fraction**	10.4 ± 3.2	$2.32\ 10^5 \pm 9.1\ 10^4$
**Precipitating fraction**	106.9 ± 7.3	$2.39\ 10^6 \pm 4.1\ 10^5$

Table S2. Most abundant corona proteins in levitating and precipitating fractions by nanoliquid chromatography MS/MS analysis. Data are reported as detected intensity normalized to molecular weight, for each of the identified proteins. Only proteins with normalized intensity > 10^6 in at least one of the investigated samples (*i.e.*, levitating and precipitating MagLev fractions) are listed (in descending order with respect to their abundance in the precipitating fraction).

	**Intensity/MW (a.u.)**	
**Protein names**	**Levitating fraction**	**Precipitating fraction**
Apolipoprotein A-I	7.38E+06	1.07E+08
Ig kappa chain C region	6.55E+06	6.36E+07
Ig lambda-6 chain C region	2.21E+06	2.54E+07
Serum albumin	3.14E+06	2.50E+07
Immunoglobulin lambda-like polypeptide 5	1.54E+06	1.71E+07
Serotransferrin	7.98E+06	1.70E+07
Keratin, type II cytoskeletal 1	1.02E+07	1.42E+07
Ig mu chain C region	8.77E+05	1.14E+07
Keratin, type I cytoskeletal 10	7.09E+06	8.29E+06
Complement C3	2.55E+05	6.87E+06
Keratin, type I cytoskeletal 9	3.06E+06	4.87E+06
Complement C1q subcomponent subunit C	1.20E+05	3.49E+06
Alpha-2-macroglobulin	4.49E+05	3.06E+06
Keratin, type II cytoskeletal 2 epidermal	2.43E+06	2.91E+06
Inter-alpha-trypsin inhibitor heavy chain H2	7.66E+04	2.71E+06
Ig kappa chain V-III region POM	6.77E+04	2.54E+06
Ig kappa chain V-III region B6	1.56E+05	2.43E+06
Immunoglobulin kappa variable 2-29	1.47E+05	2.38E+06
Complement C1q subcomponent subunit B	6.13E+04	1.93E+06
Apolipoprotein D	1.35E+05	1.91E+06
Serum amyloid A-4 protein	3.88E+04	1.88E+06
Ig kappa chain V-III region VG	1.27E+05	1.88E+06
Inter-alpha-trypsin inhibitor heavy chain H4	4.82E+04	1.71E+06
Alpha-1B-glycoprotein	6.69E+04	1.67E+06
Apolipoprotein B-100;Apolipoprotein B-48	1.96E+04	1.44E+06
Complement C1q subcomponent subunit A	5.08E+04	1.41E+06
Keratin, type I cytoskeletal 14	9.41E+05	1.28E+06
C4b-binding protein alpha chain	2.21E+04	1.27E+06
Ig lambda chain V-III region SH	3.37E+04	1.24E+06
Ceruloplasmin	1.83E+05	1.14E+06
Dermcidin	1.87E+06	1.06E+06
Complement factor H	4.82E+04	1.06E+06
Ig lambda chain V-I region HA	3.50E+04	1.05E+06
Ig kappa chain V-I region HK102	5.38E+04	1.04E+06
Inter-alpha-trypsin inhibitor heavy chain H1	4.49E+04	1.01E+06
